# An updated and unified earthquake catalog from 1787 to 2018 for seismic hazard assessment studies in Mexico

**DOI:** 10.1038/s41597-019-0234-z

**Published:** 2019-10-29

**Authors:** Rashad Sawires, Miguel A. Santoyo, José A. Peláez, Raúl Daniel Corona Fernández

**Affiliations:** 1 Institute of Geophysics - Morelia Campus, National Autonomous University of Mexico (UNAM), 58190 Morelia, Michoacán Mexico; 2 Department of Geology, Faculty of Science, Assiut University, 71516 Assiut, Egypt; 3 Department of Physics, University of Jaén, 23071 Jaén, Spain; 4 Postgraduate School in Earth Sciences, National Autonomous University of Mexico (UNAM), Mexico City, Mexico

**Keywords:** Seismology, Natural hazards

## Abstract

Here we present a new updated and unified Poissonian earthquake catalog for Mexico. The details about the catalog compilation, the removal of duplicate events, unifying the magnitude scales, removal of dependent events through the declustering process and its completeness analysis are presented. Earthquake and focal mechanism data have been compiled from various local, regional and international sources. Large earthquake events (M_W_ ≥ 6.5) have been carefully revised for their epicentral locations and magnitudes from trusted publications. Different magnitude-conversion relationships, compatible with available local and regional ones, has been established to obtain unified moment magnitude estimates for the whole catalog. Completeness periods for the declustered catalog were estimated for the definition of appropriate seismic source models for the whole territory. The final unified Poissonian earthquake catalog spans from 1787 to 2018, covering a spatial extent of 13° to 33°N and 91° to 117°W. This catalog is compatible with other published catalogs providing basis for new analysis related to seismicity, seismotectonics and seismic hazard assessment in Mexico.

## Abstract (summary)

**Table:** 

Measurement(s)	earthquake
Technology Type(s)	digital curation
Factor Type(s)	location
Sample Characteristic - Environment	planetary crust
Sample Characteristic - Location	Mexico

Machine-accessible metadata file describing the reported data: 10.6084/m9.figshare.9933536

## Background and Summary

The occurrence of seismic events in numerous regions of the world, especially those with resultant losses in human lives, have highlighted the urgent necessity of implementing specific regulations on the seismic design codes for each specific region. Long-term earthquake hazard assessment is one of the most important tools for seismic risk mitigation and the reduction of financial and life losses related to such catastrophic events. Besides that, the construction of an early warning system together with the public awareness for natural disasters are essential complementary actions.

The fundamental information necessary for any seismic hazard study is the most complete seismic record possible. This record, also termed the seismic catalog, should include at least the spatial coordinates of the epicenters, times of occurrence together with magnitudes of the earthquakes that took place in the region of interest. The quality and homogeneity of such information is reflected directly in the final seismic hazard results. Therefore, earthquake catalogs as well as focal-mechanism catalogs to provide a deep understanding of the seismotectonic setting of the area of interest are basic to develop a reliable seismic source model. A seismic source model together with a representative ground motion attenuation model considering the local site conditions are the primary components required to carry out an appropriate seismic hazard study. Instrumental earthquake catalogs show the overall seismicity of the Earth since about 1904 (e.g., ISC-GEM catalog). However, examining and inspecting the regional historical earthquakes, in addition to the instrumental recorded events, is essential to understand the long-term seismicity.

Mexico is situated in one of the most active seismic belts of the planet. Its tectonic setting is highly complex. Most of the active seismic regions in and around Mexico are related to the interaction among five tectonic plates (Supplementary Figure I). One of the most important is the subduction of the Cocos and the Rivera tectonic plates beneath the North American plate along the Middle America Trench in the southern coast of Mexico. Among the largest earthquakes that took place along the subduction boundary are the June 3, 1932 (Mw 8.2) Colima-Jalisco, the September 19, 1985 (Mw 8.06) Michoacán, the October 9, 1995 (Mw 8.0) Colima and the September 8, 2017 (Mw 8.2) Tehuantepec earthquakes. A second important plate interaction is the divergence between the Pacific and the North American plates (in northwestern Mexico), which also generates large earthquakes along the spreading center/transform boundary of the Gulf of Baja California. Large earthquakes have occurred along the spreading transform boundary, for example, the April 4, 2010 (Mw 7.2) El Mayor-Cucapah earthquake. A third significant region generating important earthquakes is the Trans-Mexican Volcanic Belt (TMVB; Supplementary Figure I), producing large crustal earthquakes, like the November 19, 1912 (M~6.9) Acambay, Mexico, earthquake. The TMVB crosses the whole country along an approximate E-W direction, and it is considered part of the Pacific Ring of Fire. Finally, other significant crustal earthquakes have occurred in the northern continental region, like the May 3, 1887 (M 7.3) Bavispe, Sonora, and the November 1, 1928 (M 6.5) Durango earthquakes. Among the mentioned events, the September 19, 1985 (Mw 8.0, Ms 8.1) Michoacán earthquake is considered as a turning point in the seismic record for Mexico (e.g.^1^, and articles within special issue^2^), while the September 8, 2017 (Mw 8.2) Tehuantepec earthquake represents the largest well-recorded recent earthquake in Mexico.

Although many regional and local catalogs exist for Mexico, they cover different time periods, use a variety of magnitude scales, and have different completeness periods. Our main objective in this work is to catalog all Poissonian independent earthquakes that occurred in the time period 1787 to 2018, and in the spatial area between 13° to 33°N, and 91° to 117°W. In the current work, we prepared a unified (in terms of moment magnitude “Mw”) Poissionian earthquake and focal mechanism catalogs for events ≥4.0 for the purpose of conducting seismotectonic and seismic hazard studies in Mexico. For this purpose, data were collected from a variety of sources, local (e.g., published well-known peer-reviewed papers and other resources), regional (e.g., Servicio Sismológico Nacional “SSN” bulletins) and international catalogs (e.g., International Seismological Centre “ISC”, United States Geological Survey “USGS” and Global Centroid Moment Tensor “CMT” bulletins) to achieve a unified magnitude scale. The initial compilation included all earthquakes having an assigned magnitude M ≥ 4.0 on any magnitude scale. A great effort and time have been consumed to review and revise the epicentral locations and magnitude values for the largest earthquakes (over M 6.5). For this, all previously-published peer-reviewed articles were collected to carefully check each earthquake.

Earthquake magnitudes from a variety of bulletins were reported in different magnitude scales. The initial compiled data comprised a total of about 84,000 earthquakes covering the entire previously-mentioned time period. One of the biggest challenges during this work was to build one standard format for the earthquake data. This is due to the huge number of compiled events that came from a variety of sources. The compiled catalog has been improved significantly by examining specifically the largest events (over Mw 6.5) for their locations and magnitudes, removing the duplicate events, and discernment of some missing parameters for the historical events. In addition, about 1,750 focal-mechanism solutions were compiled for the time period 1963 to 2016. They are mainly gathered from ISC and Global CMT bulletins supplemented by published solutions from international peer-reviewed papers. New empirical magnitude-conversion relationships between various reported magnitude scales (e.g., body-wave magnitude “Mb” to Mw, surface-wave magnitude “Ms” to Mw) were developed from ISC and USGS bulletin values to provide the unified moment (Mw^*^) magnitude estimates. Our empirical relationships were plotted against some worldwide known ones for comparison and confirmation. With the help of such derived relationships, we assigned the Mw as the unified magnitude scale for the whole catalog, which then allows us to evaluate the completeness periods for specific magnitudes for the entire catalog. Reported magnitudes (Mb, Ms, duration “MD”, local “ML”, and Mw magnitudes) are also included in the final database for researchers who might prefer to use different empirical relationships than those applied in the current study.

Foreshocks, aftershocks and earthquake swarms, as dependent events, have been identified and removed from the catalog through a declustering process using the Gardner and Knopoff^3^ approach by applying specific time and space windows for each magnitude size. Many of the initial compiled events in the catalog was identified as dependent events and removed. A total of near 5160 events (from 1787 to 2018), represent the final Poissonian earthquake catalog including only main shocks with a magnitude above or equal to Mw^*^ 4.0, covering the region with latitude ranging from 13° to 33°N and longitude ranging from 91° to 117°W. Tabulated parameters are: the origin date and time; epicentral location (longitude and latitude); depth; reported magnitudes; the final unified Mw* value; and the different codes for each parameter. Our new and updated catalog improves upon previous catalogs in terms of certainty of epicentral locations and magnitudes of the included events. This is because all original sources have been checked carefully in terms of magnitudes and locations, among other factors.

Finally, the degree of completeness for the entire catalog was evaluated for each magnitude range. The completeness analysis of the entire catalog reveals that our catalog is complete, with respect to time, for magnitudes Mw ≥ 4.0, ≥5.0 and ≥6.0 since 1990, 1990 and 1925, with activity rates of 103.3, 36.3 and 5.31 events/year, respectively. The completeness results have been interpreted in the framework of the establishment and development of the international and national seismic networks.

Our unified catalog for Mexico is compatible with the well-known worldwide catalogs. Since this catalog covers some gaps and heterogeneities observed in some previous compilations, so it will present a useful guidance for upgrading it in the future. Thus, the current revised and unified catalog provides a solid basis to use in any seismotectonic and seismic hazard assessment for the whole country or for specific regions. The final version of the catalog is provided as an electronic supplement attached to this article.

## Methods

### Catalog compilation

During the past few decades, large efforts from many different researchers and institutions has been made in order to improve specific earthquake catalogs (specially for the largest events) for specific regions and states in Mexico (e.g.^4–7^, among many others). A number of local and national catalogs using different criteria and with different characteristics, time periods, data formats, and completeness intervals have been achieved. One of the major objectives of this work for Mexico is to develop a new updated and unified earthquake catalog based on the integration of international data sources, the SSN Mexican national network and any other related earthquake bulletins.

The first step towards the unified earthquake catalog was surveying all the available national and international data sources. Next, to unify the used format for the collected data from the different bulletins. All available parameters (e.g., origin time, geographic location, reported magnitude sizes and formats, and reference code for each data provider) have been included. The initial compiled data included all earthquake data with magnitudes equal to or greater than 4.0. The compiled catalog (also considering duplicated events) included about 84,000 events. This work implied a major struggling/challenge due to the huge number of the reported earthquakes and the major differences in the data formats and quality among the data sources. The compiled data suffered from duplication, incompleteness, and errors in both the geographic locations and the focal depths. Large effort and much time were necessary to evaluate and choose the information between the different data sources (specially for events over M 6.5) and erase the duplicate earthquake records. For those historical events (before the year 1900) and instrumental large earthquakes (over M 6.5), a detailed inspection for the previously-available publications has been made to check both the most reliable location (latitude and longitude), depths and the magnitude sizes.

In the following, we are listing the different bulletins, catalogs and sources that have been used (arranged according to priority) in the compilation of our earthquake and focal mechanism catalogs.

#### Published peer-reviewed articles (for M ≥ 6.5 events)

For those historical events (before the year 1900) and instrumental large earthquakes (over M 6.5), a detailed inspection for the previously-available publications has been made to check both the most reliable location (latitude and longitude), depths and the magnitudes. The following published works have been inspected specifically for Mexican earthquakes:

- **Abe**^8^**:** He presented a catalog for large shallow events (up to 60 km depth) that took place during the period from 1904 to 1980. Magnitudes from Ms and Mb by Gutenberg^9^ and Gutenberg and Richter^10^, respectively, were reported.
- **Singh** ***et al***.^11^**:** In their research about the seismic gaps and recurrence periods of large earthquakes along the Mexican subduction zone, they presented a catalog for the largest Mexican earthquakes (Ms ≥ 7.0) that took place from 1806 to 1979. They mentioned that the epicentral locations and magnitudes of most reported events are accurate within Δ ± 0.1° for locations and M ± 0.3 for magnitudes.
- **McNally and Minster**^12^**:** During their study of the non-uniform seismic slip rates along the Middle America Trench, they included a catalog for large events (Ms ≥ 7.0) that mainly was compiled from other sources. Among these sources can be quoted the following ones: Gutenberg^13^, Figueroa^14^, Duda^15^, Kelleher *et al*.^16^, Miyamura^17^, Geller and Kanamori^18^, Kanamori and Abe^19^, and Singh *et al*.^11^.
- **Abe and Noguchi**^20,21^**:** A list of large shallow earthquakes spanning the time period from 1898 to 1917 was presented by the authors. Earthquake sizes have been expressed as M_GR_^22^, MD^15^ and Ms magnitudes. For location and origin time, they reported them from different sources, e.g., Gutenberg and Richter^22^, Gutenberg^13^ and Duda^15^.
- **Singh** ***et al***.^4,23^**:** They presented a catalog of the largest shallow earthquakes (h ≤ 65 km; Ms ≥ 5.9) spanning the time period from 1900 to 1981 and covering the spatial region from 15° to 20°N in latitude, and from 94.5° to 105.5 °W in longitude. Their catalog is compiled mainly from other sources, e.g., Gutenberg and Richter^22^, Duda^15^, Rothé^24^, Figueroa^25^, Miyamura^17^, Abe^8^, Earthquake Data Report “EDR” (USGS), Uppsala and Göttingen bulletins, and Uppsala and Göttingen Wiechert seismograms.
- **Anderson** ***et al***.^26^**:** They discussed the seismic strain release in the Mexican subduction zone (15° to 21°N, 95° to 105.5°W), depending on the estimates of the seismic moment for a revised earthquake catalog for large events that occurred in Mexico, in the period from 1806 to 1986. Both the epicentral locations and magnitudes were compiled from previously-published sources, e.g., Gutenberg and Richter^22^, Singh *et al*.^4,11^, Yamamoto *et al*.^27^, Astiz and Kanamori^28^, UNAM Seismology Group^29^, and Nishenko and Singh^30^, among others.
- **Ambraseys**^31^ **and Ambraseys and Adams**^32^**:** The first paper presented the results of the Ms computation of Central American earthquakes for the period from 1898 to 1930, while the second paper discussed the re-examination of macroseismic information for large earthquakes (≥Ms 7.0) for the same region and for the time period from 1898 to 1994. They mentioned that the locations of the more important earthquakes were revised using a combination of macroseismic information and instrumental readings.
- **Santoyo** ***et al***.^5^**:** In this work, an estimation for the center of the rupture area of 24 shallow thrust earthquakes (Ms ≥ 6.9) was presented. This estimation was mainly based on their aftershock areas, or inferred from empirical relationships, e.g., Utsu and Seki^33^ and Wells and Coopersmith^34^. This useful information has been considered in the final catalog.
- Other references have been considered for specific regions during the compilation and revision of large earthquake events (M 6.5) in this work. For example, for those events that occurred in northwestern Mexico, in the region of Baja California^6,35,36^. In addition, other references for the largest events along the Mexican subduction zone^30,37–41^ were accounted. Moreover, some global and regional catalogs^42,43^ were also considered, in addition to the previously listed sources.
- Other published information about specific earthquakes was also collected from detailed works. Examples for the studied earthquakes are the (M 8.6) March 28, 1787^44^, the Zihuatanejo (Ms 7.0) November 16, 1925^45^, the Oaxaca (Ms 8.0) January 15, 1931^46^, the Jalisco (Ms 8.2) June 3, 1932^47,48^, the Colima (M 7.5) January 30, 1973^49^, the Orizaba (Mb 6.7) August 28, 1973^50^, the Oaxaca (Mw 7.6, Ms 7.8) November 29, 1978^51,52^, the Michoacán (Mw 8.0) September 19, 1985^53^, the Zihuatanejo (M 6.6) December 10, 1994^54^, the Copala (M 7.3) September 14, 1995^55^, the Colima-Jalisco (Mw 8.0) October 9, 1995^56^, the Chiapas (Mw 7.2) October 21, 1995^57^, the Papanoa (Mw 7.3) April 18, 2014^58^, the Chiapas (Mw 8.2) September 8, 2017^59,60^, and the Morelos-Puebla (Mw 7.1) September 19, 2017^61^ earthquakes, among others.

#### The Mexican National Seismological Service (SSN)

On September 5, 1910, the Mexican government founded the SSN (http://www.ssn.unam.mx/), being member of the International Seismological Association. During this period, the SSN belonged to the Mexican National Geological Institute, under the administration of the Mexican Ministry of Mining and Promotion. From 1910 to 1923, nine German-made “Wiechert” mechanical seismological stations were installed in Tacubaya, Oaxaca, Mérida, Chihuahua, Veracruz, Guadalajara, Mazatlan, Monterrey and Zacatecas (Supplementary Figure II). Among those seismic stations, the Tacubaya Central station is nowadays still in operation, which together with the updated SSN seismological stations, embodies one of the oldest seismic networks with continuous operation in the world. The SSN Broadband Seismological Network was configured to monitor seismicity in the regions with the greatest seismic potential within the Mexican territory. Seismic stations (Supplementary Figure II) are located along the entire country, with major concentration of stations along the Pacific coast and the TMVB. The SSN network currently consists of more than 110 stations in operation. The distribution of these stations is shown in the Supplementary Figure (II). In the present study, 23,855 earthquakes (M ≥ 4.0) have been accounted from the SSN catalog, spanning from 1990 to 2018 (Supplementary Figure IIIa).

#### The ISC online bulletin

The ISC bulletin (http://www.isc.ac.uk/) reports all earthquake data in digital format from the year 1900 (by the International Seismological Summary, ISS) until the present, and it is updated periodically. It represents one of the basic international more completed and corrected seismic bulletins, in comparison with other sources. It consists of raw and revised earthquake data collected from local and national networks (about 130 agencies) all over the world. The main earthquake parameters reported in these catalogs are: origin time, hypocenter location, phase arrival-time, magnitude, focal-mechanism solutions, etc. A number of 19,930 earthquakes (M > 4.0) (Supplementary Figure IIIb) have been collected from the ISC bulletin for this work, spanning the time period from 1900 to 2018, describing them in different magnitude sizes (Mb, Ms, Mw, ML, and MD magnitudes).

#### The EHB-ISC catalog

It is a refined version from the ISC seismic bulletin. It contains revised data from 1960 to 2009 (http://www.isc.ac.uk/ehbbulletin/)^62^. The Engdahl *et al*.^62^ algorithm has been used to improve routine hypocenter determinations (particularly the depth) carried out by the ISS, the ISC, and the Preliminary Determination of Epicenters (PDE) bulletins for teleseismic events. This was before the introduction of the new location algorithm of Bondár and Storchak^63^. Magnitude sizes are expressed in the form of Mb, Ms and Mw magnitudes. Most of Mb and Ms data were taken from the ISC bulletin, while Mw was taken from the Global CMT catalog. The number of the reported events included in the EHB-ISC bulletin for Mexico is 1,516 earthquakes (Supplementary Figure IIIc).

#### The ISC-GEM Global Instrumental Earthquake Catalog

It is the result of a great effort to adapt and improve the existing earthquake data for moderate and big events (M ≥ 5.5), which serves directly to the requirements of the analysts who are interested in evaluating seismic hazard and seismic risk. It covers the time period from 1904 to 2014 (http://www.isc.ac.uk/iscgem/). The ISC-GEM catalog was funded by the Global Earthquake Model (GEM) Foundation as part of the Global Hazard Components. Its first version is the result of a long project ended in January 2013^64^. From November 2013 to December 2017, with the support of several public and commercial agencies, they worked on the Extension Project to include global events that took place after the year 2009, and smaller earthquakes (<M 6.2) in the period 1904–1959. In January 2018, they began working on the Advancement Project, that aimed to include additional source parameters for early earthquake events from scientific literature, which in turn improved magnitude determinations by identifying and addressing some reporting gaps of quality long-term stations, and add smaller earthquakes with Mw 5.0–5.5 in continental areas during the late instrumental period (1964 – present). A number of 765 earthquake events (Mw ≥ 5.5) were collected from the ISC-GEM catalog for Mexico (Supplementary Figure IIId) in the current compilation.

#### The National Earthquake Information Center (NEIC-USGS) global earthquake bulletin

This is an online earthquake bulletin (http://earthquake.usgs.gov/earthquakes/) which covers and includes earthquakes that occurred during the time period from 1932 until present (California Integrated Seismic Network; for Mexico data). Magnitude sizes in this bulletin have been presented in terms of Mb, Ms, MD and Mw magnitudes. A total number of 15,055 earthquake events (over M 4.0) have been compiled from this online bulletin to be included in our compiled catalog (Supplementary Figure IIIe).

#### The Incorporated Research Institutions for Seismology (IRIS) catalog

It aggregates earthquake data from a number of independently-operated catalogs (http://ds.iris.edu/ds/nodes/dmc/data/types/events/catalogs/), such as NEIC-USGS, ISC, ANF/ANFR (USArray Array Network Facility (ANF) & ANFR catalog indicates the final reviewed publication) and UofW (University of Washington) bulletin sources. For Mexico, IRIS catalog includes events covering the time period from 1960 until present. Magnitude sizes for this catalog have been expressed as Mb, Ms, MD, ML and Mw magnitudes. Most of the reported earthquakes for Mexico came from the ISC bulletin, the NEIC-USGS and the Mexican SSN seismic network. A total number of 22,865 events (over M 4.0) have been compiled from this catalog (Supplementary Figure IIIf).

### Catalog merging

During merging of the previously-mentioned earthquake data, collected from different catalogs and bulletins, it was crucial to avoid any possibility for earthquake duplication. The merged earthquake data has been presented displaying for each event its date (year/month/day), time (hour/minute/second), geographic location (longitude, latitude), depth (in km), and reported magnitudes (Mb, Ms, Mw, MD, and ML) (see Table 1). Different codes have been included to define the source for the magnitude sizes for each event. Duplicated earthquakes were identified based on their geographic location and date/time of the earthquake, and finally lower-priority events have been removed from the compiled catalog. This has been done by carefully inspecting the records that correspond to the same event in the obtained catalog.

**Table 1:** Magnitude scales and types in the compiled catalog for M ≥ 4.0 before unification.

Magnitude Scale	Number of events	Time Period	Maximum reported magnitude
Mw	3,375	1787–2018	8.6
Ms	7,584	1899–2017	8.2
Mb	31,134	1912–2018	7.1
MD	23,250	1989–2017	6.7
ML	27,110	1932–2017	8.2

The merging process has been performed following the same criteria in Sawires *et al*.^65–68^. Potential duplicate events displaying a difference in the origin time less than one minute and a difference in their locations less than one latitude/longitude degree have been identified. All such records that are satisfying these two conditions have been examined manually to analyze individual cases. In this regard, because the ISC bulletin uses earthquake data collected by different seismological networks all over the globe, their locations are generally considered by the user as the basis for this work. For other events not included in the ISC bulletin, the location provided to these events by a local agency is considered. However, a preference for the parameters (geographic coordinates and origin date/time) reported by local and national sources (especially for large events already studied and reported in published papers) has been taken into account rather than those come from regional or international sources.

In terms of magnitudes (Table 1), the compiled earthquake data has been described by a number of different magnitude scales. All these magnitude types have been included for the collected events, as well as a specific code assigned to each magnitude source. However, the Mw has been preferred, followed by the Ms and Mb magnitudes. In some cases, more than one Mw magnitude are available for the same earthquake but from different sources. In such cases, the value coming from the Global CMT catalog has been chosen.

### Unifying the catalog

Different magnitude scales have been considered by several researchers (e.g.^9,10,69–72^) during the past decades. ML is the earliest magnitude scale used as an instrumentally-measured estimation of the earthquake size^69^. In the 1960s, the Mb was introduced to be reported in the ISC and NEIC bulletins by the USGS and the National Oceanic and Atmospheric Administration (NOAA), in conjunction with the establishment of the World-Wide Standard Seismograph Network (WWSSN). Later, the Ms was introduced by the NEIC bulletin and it was accepted later to be used by the ISC bulletin^73^. The main problem in the application of these scales is that they saturate for large earthquakes, which leads to the underestimation of magnitude for large earthquake events. In addition to this question, their behaviors are different over the whole magnitude range^74,75^. To overcome such problems, a new non-saturating magnitude scale (Mw) was proposed by Hanks and Kanamori^71^. This scale is based on the total scalar seismic moment released during the rupture of an earthquake. Seismic moment, and thus the Mw, is mainly controlled by both the fault/rupture area, the average dislocation, and the rigidity of the medium.

In the present work, it was required to unify the magnitude scale and homogenize the earthquake catalog, as much as possible, with respect to the Mw scale. This is because the prevailing seismic hazard assessment accept only a non-saturated magnitude. A huge number of empirical relationships is presented in the literature between the Mw and other classical magnitude scales. Some of these relationships were derived from global earthquake data sets (e.g.^76–78^), and others by using earthquake records from different seismotectonic environments (e.g.^66–68,79–87^).

In this work, a number of well-established regression relationships between the different reported magnitudes and the Mw has been specifically developed. Such relationships are those established from our database after studying and comparing with other magnitude relationships (e.g.^80^) in the scientific literature. In our final catalog (see the uploaded Microsoft Excel file entitled “Earthquake catalog (1787–2018) for Mexico”^88^), the initially reported magnitude scales have been included, in addition to the final equivalent Mw*. This allows interested researchers to use other type of magnitude scales to unify the catalog, or to use directly other empirical relationships to estimate the unified magnitude.

**Fig. 1:**
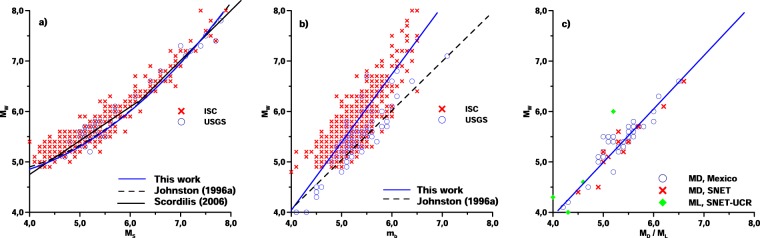
Fitting relationship between Ms-Mw, Mb-Mw, and MD/ML-Mw magnitudes.

In this work, the equivalent Mw* values were computed for each earthquake dataset from the reported magnitudes. First of all, for events that were defined originally with a reported Mw, this was finally used as the equivalent one. Second, for those earthquakes that were defined by a reported Ms magnitude, a second-degree polynomial fitting between the Ms and the Mw magnitudes (Eq. 1 and Fig. 1a) was assessed from the current catalog, using 458 events (4.0 ≤ Ms ≤ 7.9) and covering the time period from 1900 to 2017 (Table 2). The derived empirical relationship is similar to the Johnston^80^, and Scordilis^77^ equations. Then, the obtained equation was used to convert such reported Ms values to the equivalent Mw* scale. Third, for those events that were defined with the reported Mb magnitude, a linear “Ordinary Least Square OLS bisector” fitting^89^ (Eq. 2 and Fig. 1b) between the Mb and the Mw values was performed. 712 earthquakes (4.0 ≤ Mb ≤ 7.1) covering the time period from 1976 to 2017 (Table 2) were employed to assess this fitting. Finally, for earthquakes with reported mD and ML magnitudes, the OLS bisector method has been used, as in the previous case, to establish a linear relationship (Equation 3 and Fig. 1c) between both MD and ML with the Mw values. A number of 57 earthquake events (4.0 ≤ MD/ML ≤ 6.6) covering the time period from 2015 to 2017 (Table 2) has been used to develop this relationship. This relation fits jointly both MD (from SSN-Mexico and SNET) and ML data (from SNET-UCR). There is no a remarkable difference in the behavior of both used data sets. So, we applied the same relationship for all MD and ML data to be converted into Mw magnitude scale.

**Table 2:** Derived regional fitting relationships between the Ms, Mb, MD, ML magnitudes and the Mw magnitude scale.

Regression model	No. of events	Application Range	Eq.
${M}_{W}^{\ast }=\left(5.58\pm 0.29\right)-\left(0.68\pm 0.10\right){M}_{S}+\left(0.13\pm 0.01\right){M}_{S}^{2}$	458	4.0 ≤ Ms ≤ 7.9	(1)
${M}_{W}^{\ast }=\left(-1.36\pm 0.13\right)+\left(1.35\pm 0.15\right){M}_{b}$	712	4.0 ≤ Mb ≤ 7.1	(2)
${M}_{W}^{\ast }=\left(-0.31\pm 0.26\right)-\left(1.06\pm 0.21\right)\left({M}_{D}\,or\,{M}_{L}\right)$	57	4.0 ≤ MD/ML ≤ 6.6	(3)

**Fig. 2:**
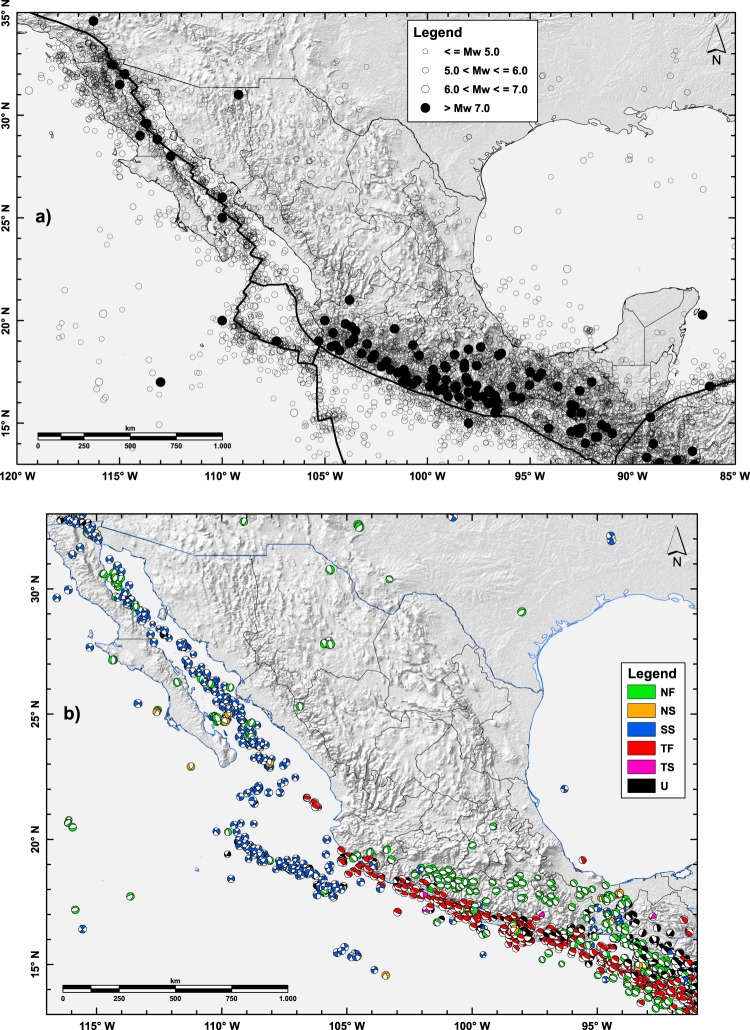
Spatial distribution of declustered main earthquakes (1787–2018) and the cataloged focal-mechanism solutions in the compiled catalog. (Green: pure normal-faulting (NF); Orange: normal-faulting with strike-slip component (NS); Blue: pure strike-slip faulting (SS); Red: pure reverse-faulting (TF); Rose: reverse-faulting with strike-slip component (TS); and Black: undefined (U)). Focal sphere sizes were plotted relative to their moment magnitudes.

In the final unified catalog (see the attached Microsoft Excel File entitled “Earthquake catalog (1787–2018) for Mexico”^88^), a specific code has been included to show the fitting relationship that has been applied to obtain the final equivalent Mw* for each reported event. The temporal distribution of the unified earthquakes included in the up-to-date catalog is plotted according to their magnitude (Supplementary Figure IVa) and number (Supplementary Figure IVb). The obtained unified catalog defined with the Mw scale has been plotted in Fig. (2a) for different magnitude ranges. Although the largest earthquakes are mainly concentrated along the plate boundaries (Fig. 2a), seismicity also occurs in other regions. The quietest seismic areas are mainly located far from the plate boundaries, towards the north and northeastern regions of Mexico.

### Catalog declustering

The spatial and temporal distribution of earthquakes is in general inhomogeneous. Computations of probabilistic seismic hazard for any region is usually based on the assumption that earthquake recurrence follows an independent distribution (memory-less process) in space and time (Poissonian distribution) (e.g.^90,91^). Therefore, foreshocks, aftershocks and seismic swarms (as dependent events) should be identified and erased through out what is called a “declustering process” since they violate the assumption of independency for earthquakes^92^. Foreshocks and aftershocks are temporally and spatially dependent on the mainshock. However, their identification is to a large degree subjective, since there are no physical differences between the foreshocks and aftershocks, as dependent events from one side, and the mainshock from the other side. As a result, earthquake clusters are typically defined by their closeness in space and time. In the declustering process, being the earthquakes arranged in space and time, the mainshock is considered as the event having the highest magnitude in a specific seismic sequence, i.e., in a specific spatial and temporal window. This process will result in a new declustered catalog containing only independent events, i.e., mainshocks.

Concerning the declustering process, there are different methodologies and algorithms that have been proposed by several researchers (e.g.^3,93–95^). The main difference among these statistical methods is the selection of the size of spatial and temporal windows, while the common factor among these methods is that the larger is the magnitude of the independent “mainshock” event, the larger is the defined spatial and temporal windows size. In this work, the dependent events have been identified and erased from the compiled catalog by using the same spatial and temporal windows parameters proposed by Gardner and Knopoff^3^. Given a certain Mw-earthquake, a full scan within a specified distance L(Mw) and time T(Mw) was performed for the whole unified catalog (e.g.^66,96,97^). Throughout this scan (see the uploaded compressed file entitled “FORTRAN CODES”^88^), the earthquake having the largest magnitude is considered to be the mainshock, and all events occurring within the L(Mw) and T(Mw) windows are declared as dependent events and erased from the catalog. Spatial and temporal window sizes of 36 km and 188 days for an Mw 4.0 event, and 100 km and 900 days for an Mw 8.0 event were used in the current declustering process. For earthquakes having in-between magnitudes, spatial (L) and temporal (T) windows sizes are computed according to the following equations:

1 $$L\left(km\right)=16{M}_{w}^{\ast }-28$$

2 $$T\left(days\right)=178{M}_{w}^{\ast }-524$$

Applying the previously-mentioned Gardner and Knopff^3^ algorithm, a total of 5,160 events are representing the final number of mainshocks (≥Mw 4.0) in the declustered catalog for Mexico, covering the spatial area between 91° and 117°W longitudes, and 13° and 33°N latitudes, during the time period from 1787 to 2018 (see the uploaded Microsoft Excel File entitled “Earthquake catalog (1787–2018) for Mexico”^88^). Magnitudes below 4.0 are not considered in the current work, due to these events are usually not included in seismic hazard studies and having a very low completeness period.

The epicentral distribution for the mainshocks has been plotted in Fig. (2a). In addition, the uploaded supplementary Microsoft Excel™ file entitled (Largest Earthquakes^88^) displays the most energetic (≥Mw 6.5) earthquakes taken place in Mexico throughout the catalog period (1787–2018). References has been included specifically for each event (for the epicentral location, magnitude and depth values).

### Focal-Mechanism solutions

Earthquake focal mechanisms are essential in seismotectonic studies. They are illustrating the relationship between earthquakes and their causative fault. Thus, they provide very useful information about the tectonic activity of the studied region. Focal-mechanism solutions for significant earthquakes that taken place in Mexico were collected mainly from the Global CMT catalog and the ISC online bulletin, as well as peer-reviewed articles (e.g.^35,98–101^).

For the Global CMT catalog, solutions are provided by Harvard University^102,103^ (http://www.globalcmt.org/). This catalog covers the time period from 1976 to 2014. All events included in this catalog are expressed using the Mw scale computed according to the Kanamori^70^ procedure. In addition, Mb and Ms magnitudes are also included for some earthquakes. A number of 784 (over Mw 4.0) solutions have been compiled from the Global CMT catalog (Fig. 2b) for the Mexican earthquakes, expressed by the two nodal planes; for each nodal plane, the strike, dip and rake values are displayed. On the other hand, for those solutions gathered from the ISC bulletin, they are aggregated mainly from a number of national and international sources (e.g., Global CMT and NEIC-USGS bulletins). A number of 1,545 solutions expressed in the Mw scale (over Mw 4.0), and covering the time period from 1963 to 2015, have been compiled for events taking place in and around Mexico (Fig. 2b).

Altogether, a number of 1,236 of events (over Mw 4.0, and from 1963 to 2015) have been obtained from both Global CMT and ISC sources, as well as published papers, after the removal of duplicated focal-mechanism solutions. An electronic supplement (see the uploaded Microsoft Excel™ file entitled “A catalog of focal mechanism solutions (1963–2015) for Mexico”^88^) has been attached to this work to show the focal mechanism solutions (values of strike, dip, and rake) for the studied events that have been collected from different sources and publications and have been plotted in Fig. (2b).

### Completeness analysis

An earthquake catalog must be as complete as possible with respect to relative frequency of the earthquake occurrence with time. Threshold or cutoff magnitude, also known as completeness magnitude (Mc) is defined as the lowest magnitude value at which all earthquakes in a specific space-time domain are reported^104^. Mc is a critical parameter in the estimation of the seismicity parameters (a- and b-values) when using the cumulative linear Gutenberg and Richter^105^ relationship. Without appropriate completeness intervals for the catalog, estimated seismicity recurrence parameters would be biased, and hence will lead to skewed estimations during the assessment of probabilistic seismic hazard. It is well known that earthquake catalogs get sparser and more uncertain once looking backward in time. In fact, completeness periods vary with time. For large earthquakes, the completeness period extends back to the pre-instrumental or the historical times, while for small-magnitude earthquakes, the completeness period is achieved only within the most recent decades of the instrumental epoch. This change in the level of completeness is mainly related to the deployment and development of the seismic networks, the increasing in the sensitivity of seismographs, and also to the significant increasing in the network coverage during the recent decades.

Identifying threshold magnitude and its spatial and temporal variations is a controversial task which does not has a single procedure to address it. The cumulative method (e.g.^106–108^) is used here for the estimating of the completeness periods. By applying such method, a simple graph is usually plotted between the cumulative number of earthquakes vs. time for a specific magnitude range (e.g., ≥Mw 4.0 or ≥Mw 6.0). The catalog is considered complete (for this particular magnitude range) with respect to time when there is approximately a straight trend (constant average slope) of the plotted data. In this case, the completeness period will be the number of years from the start of this straight-slope segment until the last year of the catalog. This method is considered to be accurate and efficient even when it is applied to a small set of earthquake data.

**Fig. 3:**
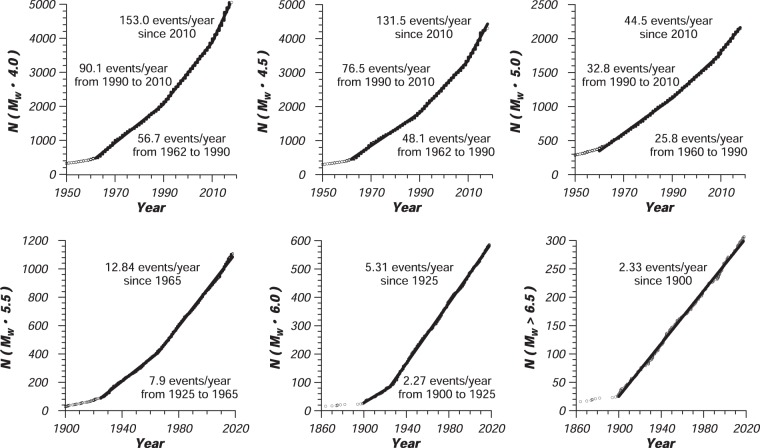
Completeness analysis for the Mexican earthquake catalog from the cumulative number of earthquakes above specific magnitude intervals.

Completeness periods and threshold magnitudes were estimated for the entire catalog. Figure (3) shows the plotting of the cumulative number of earthquakes above different magnitude levels (4.0, 4.5, 5.0, 5.5, 6.0, and 6.5) against time for the current catalog. Completeness periods for different magnitude intervals have been tabulated in Table (3). Results show that the current unified catalog is complete for magnitudes above Mw 4.0, 4.5 and 5.0 since 2010, and for magnitudes above Mw 5.5, 6.0 and 6.5 from 1965, 1925 and 1900, with seismicity rates of 153.0, 131.5, 44.5, 12.84, 5.31 and 2.33 events/year, respectively. Whereas that earthquakes above M_W_ 4.0 and M_W_ 5.0 are complete only in the last 10 years, approximately, earthquakes above M_W_ 6.0 and 6.5 are complete in the last 95 and 120 years, respectively.

**Table 3:** Results of completeness analysis for the entire catalog for Mexico (1787–2018).

Magnitude	Completeness Period	Activity Rate (events/year)
Mw ≥ 4.0	2010–2018	153.0
	1990–2010	90.1
	1962–1990	56.7
Mw ≥ 4.5	2010–2018	131.5
	1990–2010	76.5
	1962–1990	48.1
Mw ≥ 5.0	2010–2018	44.5
	1990–2010	32.8
	1960–1990	25.8
Mw ≥ 5.5	1965–2018	12.8
	1925–1965	7.90
Mw ≥ 6.0	1925–2018	5.31
	1900–1925	2.27
Mw ≥ 6.5	1900–2018	2.33

The obtained completeness periods in the current work (Table 3) appear to be in a good agreement with those values mentioned by Singh *et al*.^4^, Zúñiga *et al*.^7^ and Salgado-Gálvez *et al*.^108^. Throughout the compilation of a catalog for shallow (h ≤ 65 km) earthquakes covering the spatial region of 15° to 20°N latitudes, and 94.5° to 105.5 °W longitudes, Singh *et al*.^4^ stated that the catalog is mostly complete for earthquakes with Ms ≥ 6.5 from 1906 to 1981. Zúñiga *et al*.^7^, throughout their work about the seismotectonic regionalization of Mexico, compiled a catalog (until 2014) from the ISC bulletin, the Mexican SSN, Red Sísmica del Noroeste de México “RESNOM”, PDE, and CMT catalogs in the form of Ms magnitudes. They noticed changes during the completeness analysis of their catalog on the years 1935, 1965, 1970, 1982 and 2003. According to their work, the catalog was considered to be complete for magnitudes Mw ≥ 6.5 and 7.0 since 1935 and 1900, respectively. On the other hand, in the course of a probabilistic seismic hazard analysis for Latin America and the Caribbean, Salgado-Gálvez *et al*.^109^ assembled a catalog (from 1900 to 2015) which comes mainly from international sources^64,72,110^. They stated that, for Mexico and Central America, the catalog is complete for Mw 4.0, 4.5 and 5.5 since 1972, and for Mw 6.5 and 7.5 since 1934 and 1906, respectively^44,110^.

Some of the obtained completeness intervals directly coincide with the establishment, improvement or increase in the number of seismic stations in seismic networks locally and globally. For example, 1918 (the end of World War I), mid-1960s (the deployment and operation of the WWSSN), and mid-1990s (activation of the Comprehensive Nuclear-Test-Ban Treaty Organization). Locally, the data availability increases significantly after the large coverage of Mexican SSN on the year 1925.

## Data Records

The final obtained declustered and unified earthquake catalog obtained in the current study was uploaded in the figshare repository under the title (Earthquake catalog (1787–2018) for Mexico^88^): it is a Microsoft Excel™ worksheet consisting three sheets; the first two sheets are for the codes and references of the earthquakes, while the third sheet consists of 5160 rows organized into 25 columns. Each row describes a single main earthquake event, while each column describes the related parameters for this earthquake. The names of the columns mentioned in third Microsoft Excel™ sheet are the following:

- A: YEAR; B: MONTH; C: DAY: date type variables indicating the date of each earthquake.
- D: HOUR; E: MINUTE; F: SECOND: date type variables indicating the time of each earthquake.
- G: LONGITUDE; H: LATITUDE: double type variables (three decimal numbers) indicating the location (longitude and latitude) of each event.
- J: DEPTH: double type variable (one decimal number) indicating the depth of each individual earthquake.
- L: Mb; N: Ms1; P: Ms2; R: Mw; T:MD; V: ML: double type variables indicating the reported magnitudes (one decimal number) for the included earthquakes, they are as the following: Mb (Body-wave magnitude), Ms1 and Ms2 (Surface-wave magnitudes), Mw (Moment magnitude), MD (Duration magnitude), and ML (Local magnitude).
- I, K, M, O, Q, S, U, W (Code): numbers representing the reference(s) for each previously-mentioned parameters (I for location, K for depth, M for Mb, O for Ms1, Q for Ms2, S for Mw, U for MD, and W for ML).
- X: Mweq: double type variable (one decimal point) indicating the final/equivalent computed moment magnitude for each earthquake included in the final catalog for Mexico.
- V: Code: A number indicating the way of estimating the equivalent moment magnitude.

The second uploaded compressed file at the figshare repository under the title (FORTRAN CODES^88^) contains all the codes that were used during the declustering of the unified earthquake catalog.

The third uploaded supplementary Microsoft Excel™ file under the title of (Largest Earthquakes^88^) contains all reported and checked data about the largest earthquakes (Mw ≥ 6.5) that took place in Mexico during the period from 1787 to 2018. Largest earthquakes events were described in this file by the following columns:

- EVENT NUMBER: Earthquakes were arranged in this file from the older to the most recent.
- DATE (year – month - day): Date of the event in terms of the year, month, and day.
- TIME (hour – minute - second): Time of the earthquake in terms of hour, minutes, and seconds.
- LATITUDE, LONGITUDE, LOCATION CODE: Latitude and longitude coordinates in decimal degrees, and their corresponding reference code.
- DEPTH, DEPTH CODE: Depth of each mentioned earthquake in kilometers, and its corresponding reference code.
- REPORTED MAGNITUDES (M1/M2/M3-Type-Code): Reported original magnitudes (Mw: moment magnitude; Ms: Surface-wave magnitude; Mb: Body-wave magnitude; MD: Duration magnitude; ML: Local magnitude), within their original references.
- Mw*: Final computed equivalent moment magnitude.
- NOTE: Codes in this attached Microsoft Excel™ file indicate the same number of the references in the manuscript.

The fourth uploaded Microsoft Excel™ worksheet in the figshare repository under the title (A catalog of focal mechanism solutions (1963–2015) for Mexico^88^) represents the final compiled focal mechanism solutions mentioned in the manuscript. This file consists of only one sheet; this sheet is composed for 23 columns and 1237 rows. Each row describes a single focal mechanism solution for a certain earthquake, while each column describes the related parameters for this event. The names of the columns mentioned in this Microsoft Excel™ sheet are the following:

- A: YEAR; B: MONTH; C: DAY: date type variables indicating the date of each earthquake.
- D: HOUR; E: MINUTE; F: SECOND: date type variables indicating the time of each earthquake.
- G: LATITUDE; H: LONGITUDE: double type variables (three decimal numbers) indicating the location (latitude and longitude) of each event.
- I: Mw*: double type variable (one decimal point) indicating the final considered moment magnitude for each earthquake included in the catalog.
- J: DEPTH: double type variable (one decimal number) indicating the depth of each individual earthquake.
- K: Strike 1; L: Dip 1; M: Rake 1; N: Strike 2; O: Dip 2; P: Rake 2: these columns represent the two nodal planes for the focal mechanism solution for each earthquake; each column contains a number for each mentioned individual parameter (strike, dip, and rake angles).
- Q: Mb; S: Mw; U: Ms: double type variables indicating other reported magnitudes (one decimal number) for the included earthquakes, they are as the following: Mb (Body-wave magnitude), Mw (Moment magnitude), and Ms (Surface-wave magnitude).
- R: Mb Reference; T: Mw Reference; V: Ms Reference; W: Bulletin: these columns mentioned the references for the Mb, Mw, Ms and the source of the focal mechanism solution, respectively.

## Technical Validation

Original reported magnitudes for all earthquakes in our catalog are included in the final database as a reference for those researchers who might prefer to use other empirical relationships to unify the catalog other than those applied in the current study.

Declustering approach that has been used in the current work has been included throughout the uploaded “FORTRAN CODES” on figshare^88^, in order to give the possibility to check them or to apply another declustering algorithm for the entire catalog.

All references used during the compilation of the earthquake catalog are included as “Codes” in the final dataset, specifically for each parameter for the largest earthquakes. This allows to check event by event from their original published references and bulletins.

## Supplementary information

Supplementary Figures

## Data Availability

The input data in this work can be accessed at the following website pages: Global CMT catalog, available at http://www.globalcmt.org/ (last accessed on April 2019); ISC bulletin, available at http://www.isc.ac.uk/iscbulletin/ (last accessed on April 2019); ISC-GEM catalog, available at http://www.isc.ac.uk/iscgem/ (last accessed on April 2019); and the USGS catalog, available at http://earthquake.usgs.gov/data/centennial/ (last accessed on April 2019). The SSN data was provided by the Mexican SSN authorities by direct request. The FORTRAN CODES^88^ used for the declustering process as well as the final obtained earthquake^88^ and focal mechanism^88^ catalogs published in this study are available through the Supplementary Data Files on *figshare*.
