# Supplementary material for: An updated and unified earthquake catalog from 1787 to 2018 for seismic hazard assessment studies in Mexico
Source: Sci Data. 2019 Oct 29;6:241. doi: 10.1038/s41597-019-0234-z (PMC6820746; doi:10.1038/s41597-019-0234-z)
Supplement: Supplementary file 1 — Supplementary Figures [file 41597_2019_234_MOESM1_ESM.pdf]

Supplementary Figures

Title: An updated and unified earthquake catalog from 1787 to 2018 for seismic hazard assessment studies in Mexico

Authors: Rashad Sawires[1,2], Miguel A. Santoyo[1], José A. Peláez[3] and Raúl Daniel Corona Fernández[1,4]

Affiliations:

[1] Institute of Geophysics - Morelia Campus, National Autonomous University of Mexico (UNAM), 58190-Morelia, Michoacán, Mexico

[2] Department of Geology, Faculty of Science, Assiut University, 71516-Assiut, Egypt

[3] Department of Physics, University of Jaén, 23071-Jaén, Spain

[4] Postgraduate School in Earth Sciences, National Autonomous University of Mexico (UNAM), Mexico City, Mexico

Table of Contents

Page

Supplementary Figure I. Global tectonic sketch for the Mexican Republic and its surroundings (TMVB: Trans-Mexican Volcanic Belt)……………………….. 2

Supplementary Figure II. Geographic distribution of the seismic stations of the Mexican Broadband SSN Network………………………………………………. 3

Supplementary Figure III. Spatial distribution of cataloged earthquakes in the compiled catalog after: a) SSN, b) ISC, c) EHB-ISC, d) ISC-GEM, e) NEIC-USGS, and f) IRIS bulletins……………………………………………………… 4

Supplementary Figure IV. a) Temporal distribution (since 1900) of the earthquakes included in the final unified catalog; b) Number of earthquakes (since 1900) in the final unified catalog distributed by year……………………… 5

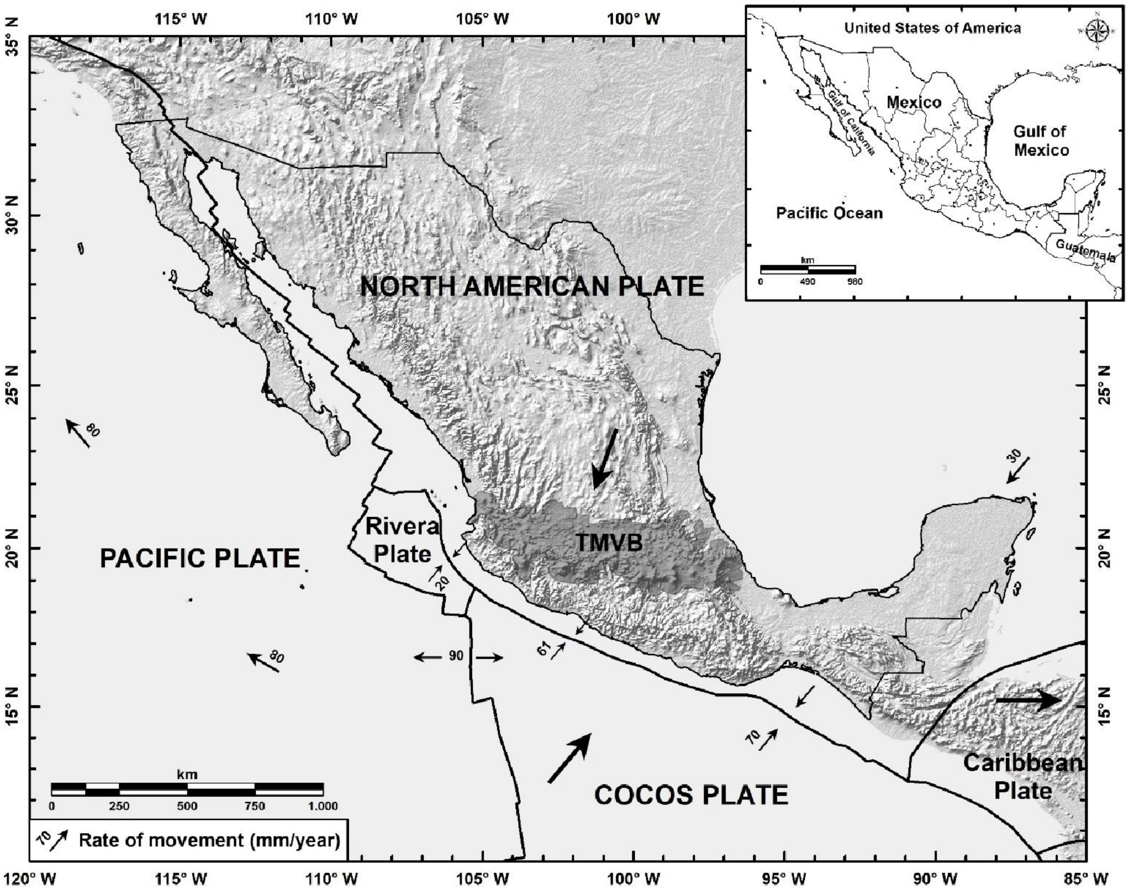

Supplementary Figure I. Global tectonic sketch for the Mexican Republic and its surroundings (TMVB: Trans-Mexican Volcanic Belt).

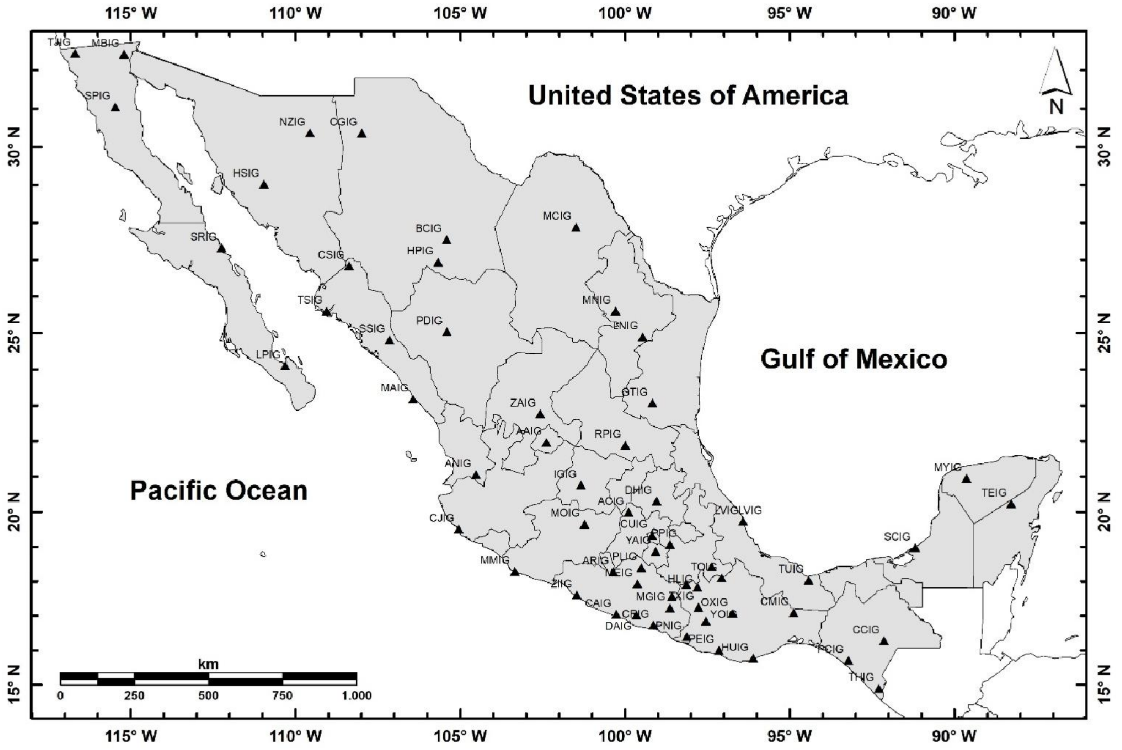

Supplementary Figure II. Geographic distribution of the seismic stations of the Mexican Broadband SSN Network.

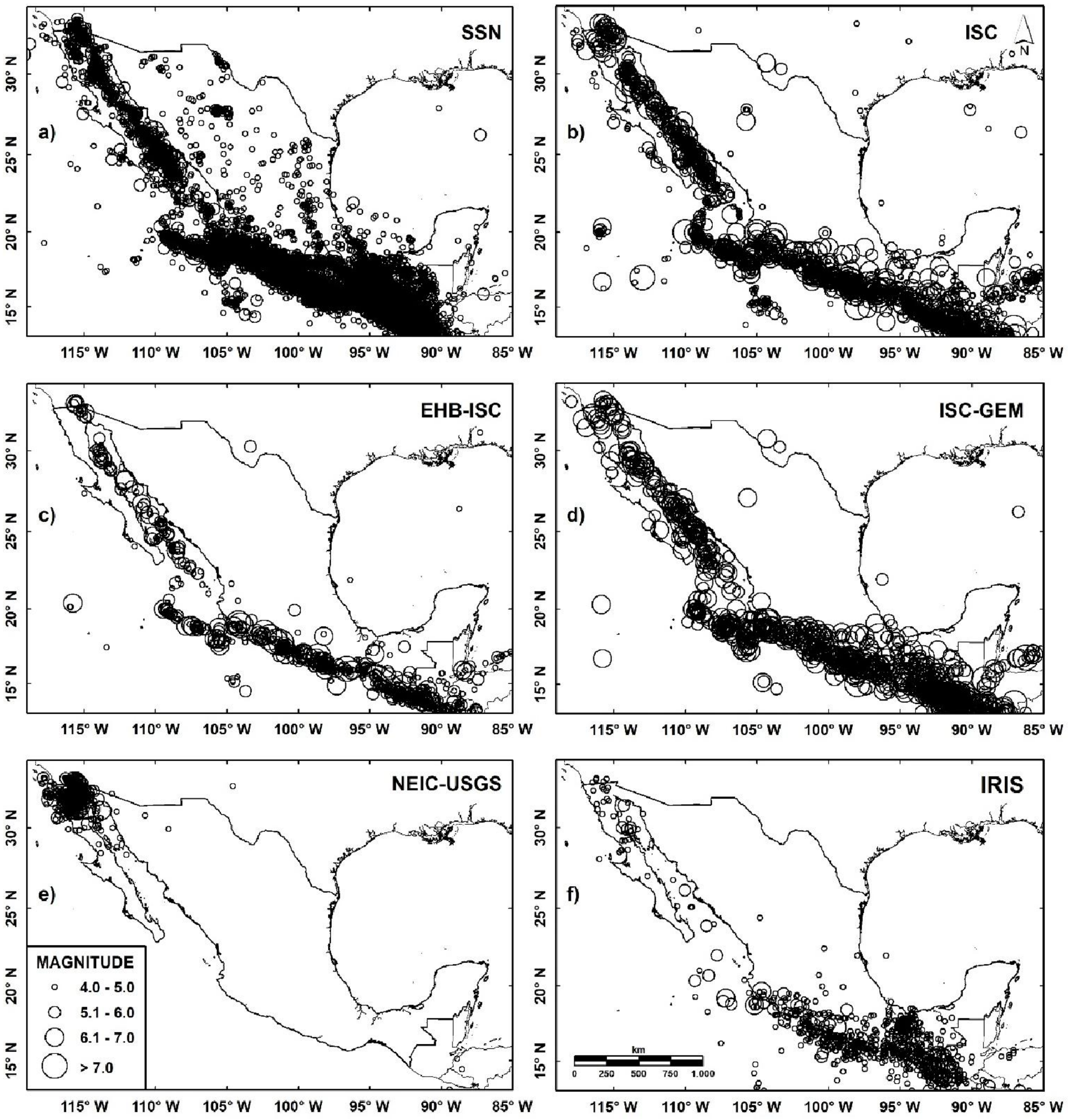

Supplementary Figure III. Spatial distribution of cataloged earthquakes in the compiled catalog after: a) SSN, b) ISC, c) EHB-ISC, d) ISC-GEM, e) NEIC-USGS, and f) IRIS bulletins.

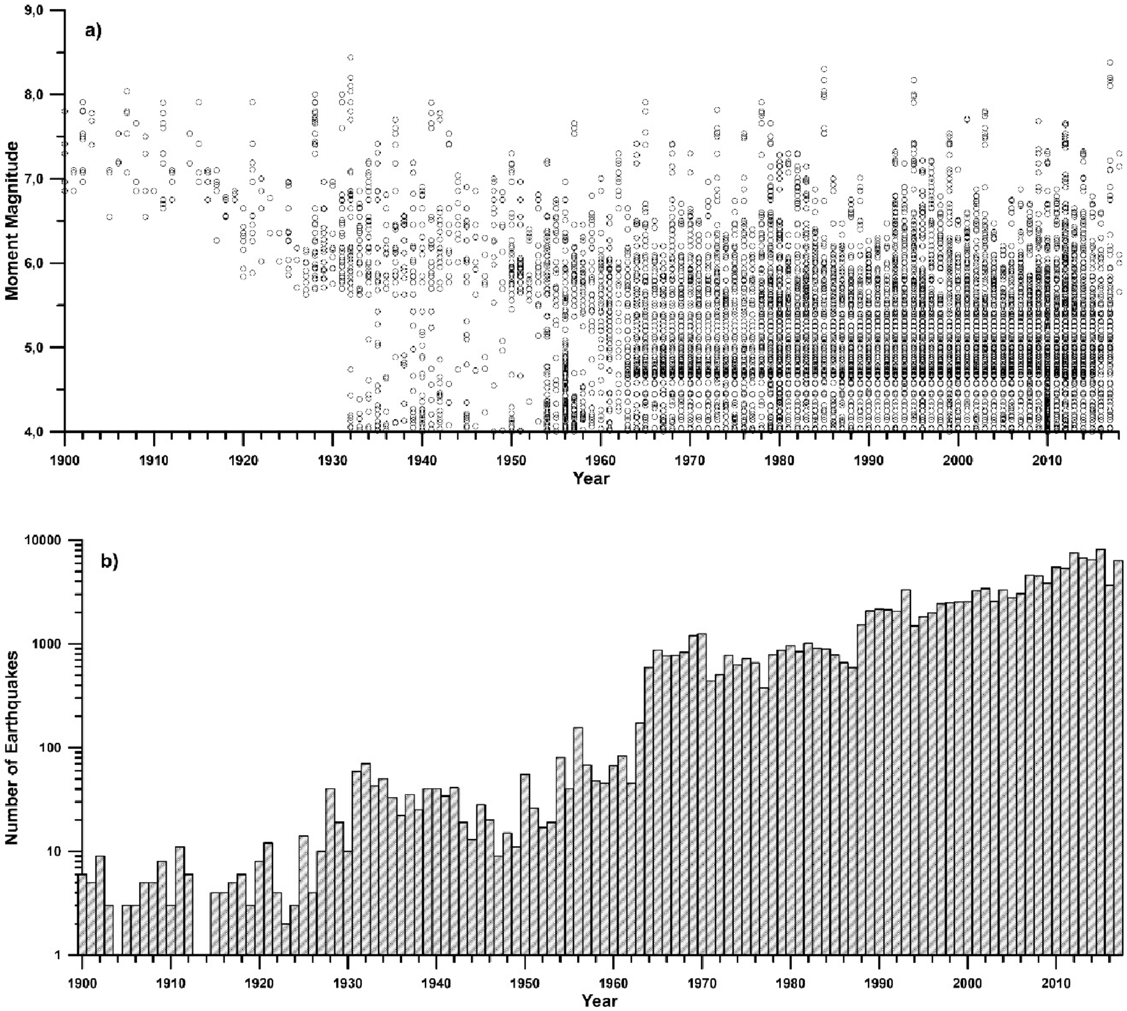

Supplementary Figure IV. a) Temporal distribution (since 1900) of the earthquakes included in the final unified catalog; b) Number of earthquakes (since 1900) in the final unified catalog distributed by year.
